# Enhancement of fibrinogen-triggered pro-coagulant activation of monocytes *in vitro *by matrix metalloproteinase-9

**DOI:** 10.1186/1477-9560-8-2

**Published:** 2010-01-29

**Authors:** Nicole C Kaneider, Birgit Mosheimer, Andrea Günther, Clemens Feistritzer, Christian J Wiedermann

**Affiliations:** 1 Division of Internal Medicine I, Department of Internal Medicine, Medical University of Innsbruck, Peter-Anich-Street 35, 6020 Innsbruck, Austria; 2 Division of Vascular Surgery, Department of Surgery, Medical University of Innsbruck, Peter-Anich-Street 35, 6020 Innsbruck, Austria; 3 Department of Internal Medicine, Central Hospital of Bolzano, Lorenz-Böhler-Street 5, 39100 Bolzano (BZ), Italy

## Abstract

**Background:**

Interaction of fibrinogen with specific leukocyte integrins of monocytes may link coagulation and inflammation, however, the precise mechanism of fibrinogen leading to the pro-inflammatory and pro-coagulatory response on monocytes is yet unknown.

**Results:**

Fibrinogen and its digestion fragment D induced pro-coagulant activation of monocytes as assessed in a cellular coagulation assay by reductions in clotting times. Pro-coagulant activation was reversed by blocking antibodies against Mac-1 or LFA-1. Pre-exposure of monocytes to the p38 MAPK inhibitor SB 202190 and the MEK1.2 inhibitor U0126 led to significant increasees in coagulation times whereas blocking JNKII with its inhibitor had no such effect. Blocking NFκB with MG-132 also inhibited pro-coagulant activation of monocytes by fibrinogen. A selective inhibitor of matrix metalloproteinase-9 increased times to clot formation whereas other matrix metalloproteinase inhibitors did not significantly interfere with fibrinogen-augmented clot formation in this assay. Treatment of monocytes with fibrinogen increased concentrations of matrix metalloproteinase-9 immunoreactivity in their supernatants.

**Conclusions:**

Fibrinogen induces monocyte pro-coagulant activation in an integrin-, nuclear factor κB-, p38 MAPK-, and MEK1.2-dependent manner. Activation of monocytes by fibrinogen increases metalloproteinase-9 secretion, metalloproteinase-9 itself enhances monocyte coagulation by an autocrine mechanism. Results provide further evidence that mediators of hemostasis have a profound impact on cells of the immune system and are closely related to inflammatory pathways.

## Background

Fibrinogen is a 45 nm long glycoprotein consisting of three pairs of polypeptide chains, Aα, Bβ and γ, symmetrically interconnected through multiple disulfide bonds forming a dimer. In addition to its well-known functions in hemostasis, over the past two decades there has been an increasing appreciation of the important function that fibrinogen exerts in the innate immune system. Studies indicate that fibrinogen plays a multifaceted role in inflammatory response, indicative of a close relationship between hemostatic and inflammatory pathways [1-4]. Acute inflammatory events are known to shift the hemostatic balance toward a pro-thrombotic state [5-7]. One established mechanism whereby inflammatory mediators can promote coagulation is the enhanced expression of tissue factor on endothelial cells and monocytes [8,9].

The ability of fibrinogen to participate in the inflammatory response depends on its interaction with specific leukocyte integrins [10-13]. The main fibrinogen receptors on leukocytes are CD11b/CD18 (Mac-1, α_m_β_2_) and CD11c/CD18 (α_x_β_2_). Leukocyte emigration from the blood to the sites of inflammation is currently viewed as an adhesion cascade that involves coordinated function of a variety of adhesion receptors on leukocytes and endothelial cells [14]. It has been shown that elevated plasma fibrinogen and fibrinogen degradation products (FgDP) inhibit several functions in neutrophils critical to the bactericidal activity of inflammatory cells [12]. Furthermore it has been suggested that fibrinogen production may be controlled by regulatory proteins produced by monocytes in response to the fibrinogen fragments D and E [15]. Conceivably FgDPs could stimulate monocytes to release interleukin-1, interleukin-6 and TNF-α[11]. Moreover fibrinogen acts as a bridging ligand for the adhesion of monocytes to cultured endothelial cells by the binding of a specific sequence of its D-domain to ICAM-1 on endothelial cells [16,17]. The N-terminal disulfide knot binds to CD11b/CD18 and CD11c/CD18 (α_x_β_2_) on stimulated neutrophils [18].

Monocytes play a key role in the orchestration of the pro-inflammatory response. These cells migrate from the peripheral blood into various tissues and differentiate into macrophages. Cells of the mononuclear phagocytotic system have been linked to a variety of inflammatory diseases, particularly to atherosclerosis, where macrophages transform into foam cells and lead to the plaque formation. Moreover, elevated fibrinogen levels in young people were independently associated with subclinical atherosclerosis [19]. Interaction of fibrinogen with specific leukocyte integrins of monocytes may link coagulation and inflammation, however, the precise mechanism of fibrinogen leading to the pro-inflammatory and pro-coagulatory response on monocytes is yet unknown.

## Results

### Pro-coagulant activation of monocytes by fibrinogen

**Figure 1:**
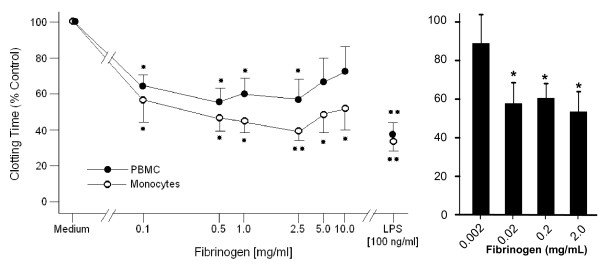
**Pro-coagulant activation of human monocytes by fibrinogen**. To test the ability of human fibrinogen to accelaerate monocyte coagulation, cells were pre-exposed to Haemocomplettan^®^, a plasma-derived fibrinogen concentrate in clinical use, (left panel) or to a commercially available fibrinogen from Haematological Technologies Inc (right panel). Both products led to a comparable inhibition of the clotting time at concentrations as low as 2 mg/mL. As endotoxin is known to induce monocyte coagulation it served as positive control. PMCs and monocytes were used for the left panel, monocytes were used in the experiments on the right panel. Normalized results are given as mean ± SEM, n = 6. Time to clot formation of control monocytes (RPMI 1640 treated) was 360 ± 46.7 sec. Statistics were calculated using Kruskal Wallis and Mann Whitney U test. *: p < 0.05.

In order to assess fibrinogen's potential to form stable monocyte conglomerates, coagulation assays were performed. Clotting time of cells pre-incubated with either lipopolysaccharide (LPS) or interleukin-1 (IL-1) was 60% reduced compared to control (RPMI 1640-) treated cells (Fig. 1; IL-1 data not shown). Treatment with fibrinogen, either Haemocomplettan^® ^ or control fibrinogen (Haematological Technologies Inc.) reduced the clotting time of monocytes. At its most potent concentration of 2 mg/mL, Haemocomplettan^® ^and control fibrinogen reduced the time for clot formation up to 60% and 45%, respectively, compared to untreated cells.

**Figure 2:**
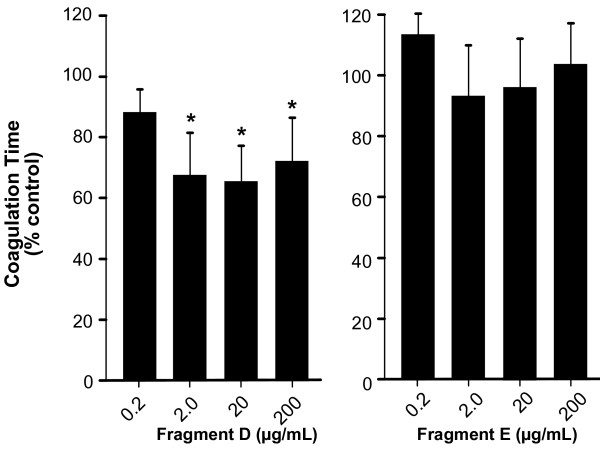
**Pro-coagulant activation of human monocytes by fibrinogen fragments D and E**. The biological activity of the fibrinogen digestion fragments D and E were tested. Cells were pre-treated for 4 h under rotating conditions with fibrinogen fragments D or E (0.2 mg/mL) and the procoagulant activity of monocytes was tested. Like the entire molecule, fragment D decreased coagulation time significantly to 65% of untreated control cells (348 ± 56.9 sec) whereas fragment E had only a non-significant effect on the formation of stable monocyte clots. Results are given as mean ± SEM, n = 3.*: p < 0.05.

As fibrinogen is usually digested into its fragments D and E in vitro, we tested the effects of these fragments on the coagulation time of human monocytes. Only fragment D was able to induce clot formation, while fragment E had no appreciable effect on clot formation (Fig. 2).

**Figure 3:**
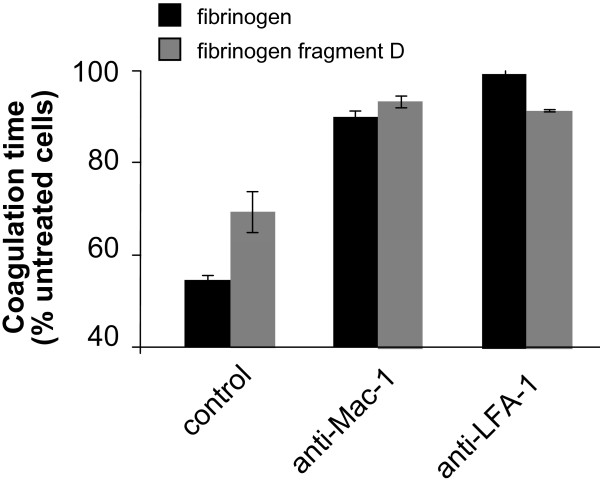
**Effect of blocking antibodies against the major leukocyte integrins on fibrinogen-triggered pro-coagulant activation of human monocytes**. Pre-treatment of human monocytes in the additional presence of blocking antibodies against Mac-1 or LFA-1 led to a sustainable prolongation of the coagulation time compared to fibrinogen- or digestion fragment D-treated cells alone; antibodies in the absence of fibrinogen or fibrinogen fragment D did not alter coagulation times (data not shown). Experiments were performed with the intact fibrinogen molecule. Results are given as mean ± SEM, n = 3. Control: p < 0.05.

Earlier reports have indicated that monocytes which are pre-stimulated with TNF-α adhere to the E- fragment whereas unstimulated monocytes only adhere to the D fragment and the N-terminal disulfide knot [11]. However, different domains of the fibrinogen molecule seem to interact with different integrins [11]. One possible explanation for the difference in monocyte reactivity to the different digestion products might be related to differences in integrin expression on leukocytes [11]. Therefore coagulation experiments with blocking antibodies directed against the most important leukocyte integrins, LFA-1 and Mac-1, were performed. Interestingly, blockade of both inegrins, LFA-1 and Mac-1, resulted in prolonged coagulation times compared to fibrinogen-only-treated cells. This observation was true for intact fibrinogen molecules as well as the D-fragment (Fig. 3). Blockade of integrins without fibrinogen exposure failed to increase the time to clot formation (data not shown).

**Figure 4:**
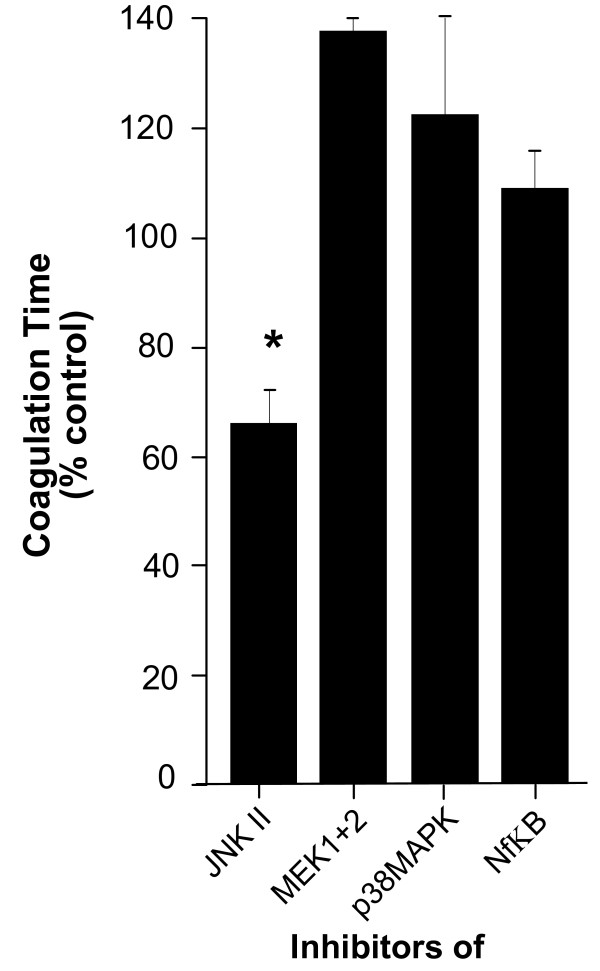
**Inhibitors of the p38 MAPK and MEK1/2 pathway block fibrinogen-triggered pro-coagulant activation of human monocytes**. As integrins primarly signal via p38 MAPK and MEK1/2 pathways, experiments with inhibitors against p38 MAPK, MEK 1/2 and JNKII were performed. Pre-exposure of the monocytes to the p38 MAPK inhibitor SB 202190 and the MEK 1.2 inhibitor U0126 led to a significant increase in coagulation time whereas blocking JNKII with its inhibitor had no effect on coagulation. Activation of NFκB by certain stimuli switches monocytes from their resting state into activated monocytes that transform into macrophages. Blocking NFκB with MG-132 inhibited the activation of monocytes by fibrinogen, the pro-coagulant activity of MG-132-treated cells was 107% of un-treated control cells. Results are given as mean ± SEM, n = 3.*: p < 0.05.

Usually, integrin-activation results in the activation of the ERK/MAPK pathway [20]. To further assess the importance of integrin signaling in the interaction of fibrinogen with monocytes, we performed signal transduction experiments with inhibitors of the ERK/MAPK pathway. Inhibitors of p38 MAPK and MEK1/2 reversed fibrinogen-induced clotting of monocytes whereas the inhibition of JNKII had no effect on coagulation times (Fig. 4). Furthermore, inhibition of NF-κB delayed clot formation, suggesting that pro-inflammatory pathway activation might be required to increase the capacity of monocytes to form aggregates.

### Matrix metalloproteinase-9-mediated activation of monocytes by fibrinogen

**Figure 5:**
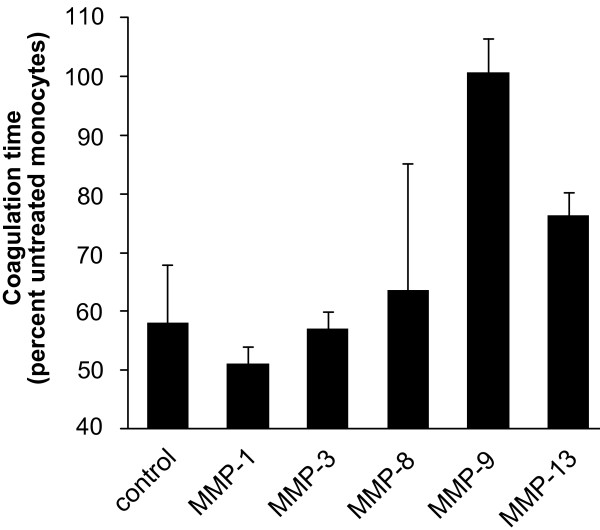
**Inhibition of MMP-9 decreases the fibrinogen-triggered pro-coagulant activation of human monocytes**. Monocytes were pre-treated with specific inhibitors of matrix metalloproteases (MMP)-1, -3, -8, -9 and -13, and exposed to fibrinogen. Then coagulation experiments were performed. The inhibitor of MMP-9 increased the time to clot formation to 355 ± 23.4 sec which was about the same for un-treated cells. Other MMP inhibitors did not interfere with fibrinogen-initiated clot formation. Results are given as mean ± SEM, n = 3. MMP-9: p < 0.05.

The major ligand-binding site within the α-chain of leukocyte integrins is called inserted domain (I-domain) and is homologous to the A domains of von Willebrand factor [12]. Interestingly, matrix metalloproteinase-9 (MMP-9), the most abundant MMP produced by monocytes, also binds to this I-domain of integrins and initiates the adhesion process [13]. Therefore, we performed cellular coagulation experiments in the presence of MMP-inhibitors. Again, cells were activated by fibrinogen and co-treated with MMP-inhibitors. Pre-treatment with a MMP-9 inhibitor completely reversed fibrinogen-induced clot formation of monocytes, whereas multiple other MMP inhibitors directed towards MMP-1, MMP-3, MMP-8, and MMP-13, did not affect monocyte aggregation (Fig. 5). Inhibition of various MMPs did not influence the coagulation time of untreated monocytes (data not shown).

**Figure 6:**
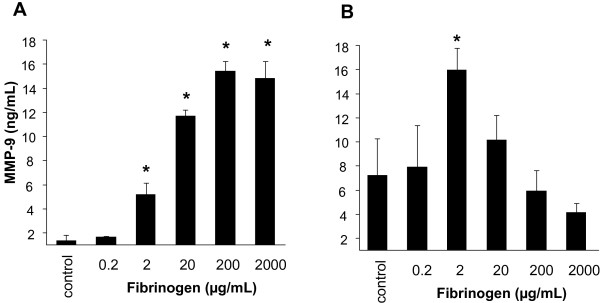
**Fibrinogen-treatment induces MMP-9 secretion from human monocytes**. Human monocytes were exposed to different concentrations of fibrinogen and MMP-9 levels were measured in the supernatants (A) and intra-cellularly (B). In the supernatants of the fibrinogen-treated cells, MMP-9 levels were 15 times higher than in un-treated monocytes were measured. Intracellular MMP-9 levels did not increase twice the concentration of untreated cells. Results are given as mean ± SEM, n = 3.*: p < 0.05.

Monocytes stimulated either by fibrinogen or other pro-inflammatory mediators may exert an autocrine mechanism of integrin activation by the further production and release of MMP-9. Therefore release into the supernatants of fibrinogen-activated monocytes of MMP-9 and, as a control, MMP-1 was tested, as well as were the intracellular levels of these MMPs measured. There was no difference in MMP-1 levels after fibrinogen treatment (data not shown). However, extra-cellular MMP-9 levels increased concentration-dependently up to 15-fold as compared to supernatants of untreated cells (Fig 6A). Intra-cellular MMP-9 levels showed a different pattern: upon stimulation with fibrinogen at the high concentration of 2 mg/mL, intra-cellular MMP-9 content dereased to lower levels as compared to stimulation of cells with the lower concentration of 2 μg/mL (Fig 6B). The reason for this phenomenon might be enhanced secretion of the metalloproteinase in monocytes with fibrinogen at higher concentrations. At lower concentrations release into supernatants and de novo production might be in steady state.

## Discussion

In this investigation, we provide evidence that fibrinogen-induced clot formation of monocytes dependents on Mac-1 and LFA-1 activation. Fibrinogen is thought to bind to leukocyte integrins and causes leukocyte adherence to the injured vessel wall. In this regard, recent studies demonstrated that simultaneous fibrinogen binding to leukocytes and endothelial cells enhances adhesion of monocytes to the endothelium by acting as a molecular bridge between the two cell types [16,17,21]. Stimulation of integrins by specific agonists activates and renders them competent to bind soluble fibrinogen, while non-stimulated integrins, however, are able to bind immobilized fibrinogen [12]. In our case no immobilized fibrinogen was used but all experiments were performed under rotating, non-static conditions. Therefore it is very likely that mediators other than fibrinogen led to the monocyte-monocyte adhesions observed in the coagulation experiments. Our data indicate that fibrinogen initially activates pro-inflammatory pathways since blockade of NfkB prolonged the time for clot formation. It is known that fibrinogen does not only lead to adhesion of monocytes, it also induces de novo synthesis of IL-6 and TNF-α [1].

A strong candidate for a possible monocyte-monocyte interaction via the integrin pathway was MMP-9. Interestingly, only pharmacological inhibitors of MMP-9 increased coagulation time almost to baseline levels, suggesting an important role of MMP-9 in this context. Stimulation of monocytes with fibrinogen under rotation led to a significant increase in intra- and extracellular levels of the protease. However, MMP-9 not only binds to integrins and induces adhesion, it might also augment the pro-inflammatory response to fibrinogen via a positive feedback loop.

In vitro studies have shown that fibrinogen may profoundly alter leukocyte functions leading to increased cell migration, phagocytosis, NfkB-mediated transcription and other processes [10,22,23].

## Conclusions

*In vitro*, fibrinogen induces monocyte pro-coagulant activation in an NFκB- and MMP-9-dependent manner by specific ligation of signal-transducing integrins that also induces TF expression in the cells. These results provide further evidence that mediators of hemostasis have a profound impact on cells of the immune system and are closely related to inflammatory pathways in which MMPs play a prominent regulatoy role.

## Methods

### Materials

Hanks balanced salt solution (HBSS) without Ca^2+ ^and Mg^2+ ^was purchased from Gibco BRL, Life Technologies (Vienna, Austria). Lymphoprep was from Nycomed Pharma (Oslo, Norway), anti-human CD14 antibody was from Miltenyi Biotec (Bergisch Gladbach, Germany), Bovine serum albumin (BSA), was purchased from Dade-Behring (Marburg, Germany). RPMI 1640 was purchased from Biological Industries (Kibbutz Beit Haemek, Israel). Fibrinogen was either from CSL Behring (Haemocomplettan) or from Haematological Technologies Inc. (Essex Junction, VT). The D- and E-fragment of fibrinogen were obtained from Haematological Technologies Inc as well. The E. coli LPS was from Sigma Aldrich. The signal transduction inhibitors SB 202190, U0126, JNK Inhibitor II and the NfkB MG-132 inhibitor were from Calbiochem (Gibbstown, NJ) as were the MMP-inhibitors. MMP-ELISA kits (proMMP-1 and MMP-9) and the blocking integrin-antibodies were purchased from R&D systems (Minneapolis, MN).

### Preparation of human monocytes

Mononuclear cells were prepared from peripheral venous blood (anticoagulated with EDTA) of healthy volunteers. After Lymphoprep^® ^density gradient centrifugation, peripheral blood mononuclear cells were collected and washed three times with normal saline. Positive selection of monocytes was performed by adding MACS colloidal superparamagnetic microbeads conjugated with monoclonal anti-human CD14 antibodies to cooled, freshly prepared peripheral blood mononuclear cell preparations in MACS buffer (PBS with 5 mM EDTA and 0.5% bovine serum albumin) according to the manufacturer's instructions. Cells and microbeads were incubated for 15 min at 4-6°C. In the meantime, the separation column was positioned in the MACS magnetic field and washed with MACS buffer at room temperature. The cells were washed with MACS buffer, resuspended, and loaded onto the top of the separation column. The eluent containing CD14^- ^cells was withdrawn and after removal of the column from the magnet, trapped monocytes (CD14^+^) were eluted with the six-fold amount of cold MACS buffer, centrifuged, and resuspended in medium containing 0.5% BSA.

### Monocyte coagulation assay

Based on the concept that tissue factor bearing cells localize and form the hemostatic plug, cell based models of coagulation have been developed that mimic - at least in part - *in vivo *coagulation reactions.

For the coagulation experiments, monocytes were incubated with the particular reagent for 4 h under continuous rotation to prevent monocyte adhesion. After washing 200 μL of re-calcified human plasma was added to the monocytes. Clotting time was measured in duplicate by using a coagulometer (Amelung; Lemgo, Germany). In some experiments monocytes were pre-treated with signal transduction inhibitors such as SB 202190 that inhibits p38 MAPK, U0126 which inhibits MEK1 and MEK2. Furthermore NF-kB was inhibited by the addition of MG-132, and JNK II was inhibited by the addition of JNK Inhibitor II. The concentration used was 3 × IC_50 _for each inhibitor. After washing fibrinogen was added and the cells were incubated with fibrinogen for another 4 hours.

For mouse studies animals were injected with 20 mg of fibrinogen. Four hours after injection, blood was collected by terminal cardiac puncture and the red blood cells were removed by lysis. Again, clotting time was measured by the method described above.

### Determination of MMP levels

Matrix metallo proteases (MMPs) were measured either in monocyte supernatants or after treating monocytes with lysing buffer (intra-cellular levels). Monocytes were stimulated with fibrinogen for 4 h then the supernatans were collected and subjected to commercially available proMMP-1 and MMP-9 ELISA assays. After collecting the supernatants, cells were lysed and after spinning down the cell detritus, intra-cellular MMP-levels were measured as well by the same method. section.

### *In vivo *monocyte migration assay

Six to 8 week old female CD-1 mice were given 20 mg of fibrinogen i.p. 4 days after the injection mice were sacrificed by CO_2_-inhalation and peritoneal lavage was performed with 10 mL of lukewarm 0.9% sodium chloride. Cells were counted in a Neubauer chamber. Peripheral leukocytes were counted in a Neubauer chamber after the lysis of red blood cells.

### Statistics

Results are given as mean and standard error of the mean. Statistics were calculated after Kruskal Wallis and Mann Whitney U tests using StatView (Abacus, Berkeley, CA).

## Competing interests

The authors declare that they have no competing interests.

## Authors' contributions

NCK drafted the manuscript, carried out in vitro and an animal studies. BM, AG and CF carried out experimental studies. CJW conceived of the study, and participated in its design and coordination and helped to draft the manuscript. All authors read and approved the final manuscript.

## References

[B1] Jensen T Kierulf P Sandset PM Klingenberg O Joo GB Godal HC Skjonsberg OH Fibrinogen and fibrin induce synthesis of proinflammatory cytokines from isolated peripheral blood mononuclear cells Thromb Haemost 2007 97 822 829 17479194 10.1160/th07-01-0039

[B2] Smiley ST King JA Hancock WW Fibrinogen stimulates macrophage chemokine secretion through toll-like receptor 4 J Immunol 2001 167 2887 2894 11509636 10.4049/jimmunol.167.5.2887

[B3] Sitrin RG Pan PM Srikanth S Todd RF Fibrinogen activates NF-kappa B transcription factors in mononuclear phagocytes J Immunol 1998 161 1462 1470 9686612

[B4] Rubel C Fernandez GC Rosa FA Gomez S Bompadre MB Coso OA Isturiz MA Palermo MS Soluble fibrinogen modulates neutrophil functionality through the activation of an extracellular signal-regulated kinase-dependent pathway J Immunol 2002 168 3527 3535 11907115 10.4049/jimmunol.168.7.3527

[B5] Esmon CT The regulation of natural anticoagulant pathways Science 1987 235 1348 1352 10.1126/science.3029867 3029867

[B6] Creasey AA Chang AC Feigen L Wun TC Taylor FB Hinshaw LB Tissue factor pathway inhibitor reduces mortality from Escherichia coli septic shock J Clin Invest 1993 91 2850 2860 10.1172/JCI116529 8514893 PMC443354

[B7] Esmon CT Taylor FB Snow TR Inflammation and coagulation: linked processes potentially regulated through a common pathway mediated by protein C Thromb Haemost 1991 66 160 165 1833850

[B8] Drake TA Ruf W Morrissey JH Edgington TS Functional tissue factor is entirely cell surface expressed on lipopolysaccharide-stimulated human blood monocytes and a constitutively tissue factor-producing neoplastic cell line J Cell Biol 1989 109 389 395 10.1083/jcb.109.1.389 2663880 PMC2115486

[B9] Drake TA Morrissey JH Edgington TS Selective cellular expression of tissue factor in human tissues. Implications for disorders of hemostasis and thrombosis Am J Pathol 1989 134 1087 1097 2719077 PMC1879887

[B10] Flick MJ Du X Degen JL Fibrin(ogen)-alpha M beta 2 interactions regulate leukocyte function and innate immunity in vivo Exp Biol Med (Maywood) 2004 229 1105 1110 15564436 10.1177/153537020422901104

[B11] Forsyth CB Solovjov DA Ugarova TP Plow EF Integrin alpha(M)beta(2)-mediated cell migration to fibrinogen and its recognition peptides J Exp Med 2001 193 1123 1133 10.1084/jem.193.10.1123 11369784 PMC2193326

[B12] Ugarova TP Yakubenko VP Recognition of fibrinogen by leukocyte integrins Ann N Y Acad Sci 2001 936 368 385 11460493 10.1111/j.1749-6632.2001.tb03523.x

[B13] Stefanidakis M Bjorklund M Ihanus E Gahmberg CG Koivunen E Identification of a negatively charged peptide motif within the catalytic domain of progelatinases that mediates binding to leukocyte beta 2 integrins J Biol Chem 2003 278 34674 34684 10.1074/jbc.M302288200 12824186

[B14] Kaneider NC Leger AJ Kuliopulos A Therapeutic targeting of molecules involved in leukocyte-endothelial cell interactions FEBS J 2006 273 4416 4424 10.1111/j.1742-4658.2006.05441.x 16956369

[B15] Ritchie DG Levy BA Adams MA Fuller GM Regulation of fibrinogen synthesis by plasmin-derived fragments of fibrinogen and fibrin: an indirect feedback pathway Proc Natl Acad Sci USA 1982 79 1530 1534 10.1073/pnas.79.5.1530 6461860 PMC346008

[B16] Languino LR Duperray A Joganic KJ Fornaro M Thornton GB Altieri DC Regulation of leukocyte-endothelium interaction and leukocyte transendothelial migration by intercellular adhesion molecule 1-fibrinogen recognition Proc Natl Acad Sci USA 1995 92 1505 1509 10.1073/pnas.92.5.1505 7878009 PMC42548

[B17] Languino LR Plescia J Duperray A Brian AA Plow EF Geltosky JE Altieri DC Fibrinogen mediates leukocyte adhesion to vascular endothelium through an ICAM-1-dependent pathway Cell 1993 73 1423 1434 10.1016/0092-8674(93)90367-Y 8100742

[B18] Lishko VK Podolnikova NP Yakubenko VP Yakovlev S Medved L Yadav SP Ugarova TP Multiple binding sites in fibrinogen for integrin alphaMbeta2 (Mac-1) J Biol Chem 2004 279 44897 44906 10.1074/jbc.M408012200 15304494

[B19] Minson CT Green DJ Measures of vascular reactivity: prognostic crystal ball or Pandora's box? J Appl Physiol 2008 105 398 399 10.1152/japplphysiol.90741.2008 18556427

[B20] Canobbio I Reineri S Sinigaglia F Balduini C Torti M A role for p38 MAP kinase in platelet activation by von Willebrand factor Thromb Haemost 2004 91 102 110 14691575 10.1160/TH03-02-0083

[B21] Altieri DC Duperray A Plescia J Thornton GB Languino LR Structural recognition of a novel fibrinogen gamma chain sequence (117-133) by intercellular adhesion molecule-1 mediates leukocyte-endothelium interaction J Biol Chem 1995 270 696 699 10.1074/jbc.270.2.696 7822297

[B22] Flick MJ La Jeunesse CM Talmage KE Witte DP Palumbo JS Pinkerton MD Thornton S Degen JL Fibrin(ogen) exacerbates inflammatory joint disease through a mechanism linked to the integrin alphaMbeta2 binding motif J Clin Invest 2007 117 3224 3235 10.1172/JCI30134 17932565 PMC2000806

[B23] Adams RA Bauer J Flick MJ Sikorski SL Nuriel T Lassmann H Degen JL Akassoglou K The fibrin-derived gamma377-395 peptide inhibits microglia activation and suppresses relapsing paralysis in central nervous system autoimmune disease J Exp Med 2007 204 571 2582 10.1084/jem.20061931 17339406 PMC2137908

